# Insertion of Tetracysteine Motifs into Dopamine Transporter Extracellular Domains

**DOI:** 10.1371/journal.pone.0009113

**Published:** 2010-02-09

**Authors:** Deanna M. Navaroli, Haley E. Melikian

**Affiliations:** 1 Interdisciplinary Graduate Program, University of Massachusetts Medical School, Worcester, Massachusetts, United States of America; 2 Department of Psychiatry, Brudnick Neuropsychiatric Research Institute, University of Massachusetts Medical School, Worcester, Massachusetts, United States of America; Institut Européen de Chimie et Biologie, France

## Abstract

The neuronal dopamine transporter (DAT) is a major determinant of extracellular dopamine (DA) levels and is the primary target for a variety of addictive and therapeutic psychoactive drugs. DAT is acutely regulated by protein kinase C (PKC) activation and amphetamine exposure, both of which modulate DAT surface expression by endocytic trafficking. In order to use live imaging approaches to study DAT endocytosis, methods are needed to exclusively label the DAT surface pool. The use of membrane impermeant, sulfonated biarsenic dyes holds potential as one such approach, and requires introduction of an extracellular tetracysteine motif (tetraCys; CCPGCC) to facilitate dye binding. In the current study, we took advantage of intrinsic proline-glycine (Pro-Gly) dipeptides encoded in predicted DAT extracellular domains to introduce tetraCys motifs into DAT extracellular loops 2, 3, and 4. [^3^H]DA uptake studies, surface biotinylation and fluorescence microscopy in PC12 cells indicate that tetraCys insertion into the DAT second extracellular loop results in a functional transporter that maintains PKC-mediated downregulation. Introduction of tetraCys into extracellular loops 3 and 4 yielded DATs with severely compromised function that failed to mature and traffic to the cell surface. This is the first demonstration of successful introduction of a tetracysteine motif into a DAT extracellular domain, and may hold promise for use of biarsenic dyes in live DAT imaging studies.

## Introduction

DA reuptake mediated by DAT is the primary means for clearing synaptic DA and terminating dopaminergic neurotransmission [1], [2]. DAT is member of the Na^+^/Cl^−^ -dependent SLC6 symporter gene family and is potently inhibited by addictive psychostimulants, such as amphetamine and cocaine, as well as by therapeutic agents such as the NDRI class of antidepressants and methylphenidate (Ritalin) [3], [4], [5]. Transgenic mouse studies indicate that DAT is critical in maintaining normal dopaminergic neurotransmission and synaptic tone, and that even a 50% reduction in DAT protein (DAT +/− mice) is sufficient to significantly alter DA signaling and synaptic stores [6], [7]. Thus, cellular mechanisms that modulate DAT cell surface expression are likely to have a significant impact on DA availability and signaling in the brain.

DAT is acutely downregulated by PKC activation [8], [9] and amphetamine exposure [10], [11], [12], which markedly decrease DAT surface levels via endosomal trafficking. While it is well established DAT surface levels are modulated by endocytic trafficking, the cellular mechanisms that mediate DAT trafficking are not well defined. Live cellular imaging is a highly effective means to reveal cellular trafficking mechanisms that are not detectable by fixed cell imaging or biochemical approaches [13], [14]. Recent studies using GFP-tagged DAT [15] and fluorescently-labeled cocaine analogs [16] have proved successful in achieving live DAT trafficking images; however, each approach has inherent limitations. GFP-tagged DAT may not behave identically to the wildtype DAT, and has the disadvantage of labeling the entire cellular DAT population, limiting the ability to examine DAT surface dynamics specifically. Use of cocaine analogs allows for exclusive labeling of the DAT surface pool, but may not reflect native DAT trafficking as cocaine binding has itself been reported to alter DAT surface expression [17], [18].

The use of biarsenic dyes, such as FlAsH, ReAsH [19] and SplAsH [20], is another potential means to fluorescently label proteins for live imaging. In particular, sulfonated versions of these dyes are membrane impermeant and may be suitable to exclusively label surface proteins [21]. Biarsenic dyes are not fluorescent in solution and bind with high affinity to tetracysteine (tetraCys) motifs, preferably flanking a proline-glycine (Pro-Gly) dipeptide (CCPGCC) [21]. Upon binding, they become highly fluorescent. In the current study, we engineered tetraCys motifs flanking existing extracellular Pro-Gly dipeptide residues encoded in human DAT (hDAT) at amino acid positions 194, 288 and 387, to use as potential biarsenic dye labeling sites. Analysis of these DAT mutants reveals that inserting a tetraCys site into DAT extracellular loop 2 (EL2) is well tolerated by DAT, and yields a functional transporter that retains sensitivity to PKC activation, whereas insertion of tetraCys into either extracellular loop 3 or 4 (EL3, EL4) perturbs DAT biosynthesis and trafficking to the cell surface.

## Methods

### Constructs and Mutagenesis

TetraCys motifs flanking existing extracellular Pro-Gly residues were engineered into EL2, EL3 or EL4 DAT extracellular loops by mutating the two residues upstream and downstream of the Pro-Gly residues into cysteines. The amino acids mutated to cysteines were: EL2: 192–193/196–197; EL3: 286–287/290–291; EL4 385–386/389–390. hDAT pcDNA3.1(+) was mutated using the QuikChange Mutagenesis kit (Stratagene). The following mutagenic primers spanning the indicated hDAT cDNA regions were used:

### EL2-CCPGCC (577–627)

5′CCCCAACTGCTCGGATTGCTGTCCTGGTTGCTGCAGTGGAGACAGCTCGG3′ (sense), 5′CCGAGCTGTCTCCACTGCAGCAACCAGGACAGCAATCCGAGCAGTTGGGG3′(antisense);

### EL3-CCPGCC (860–907)

5′CTCCTGCGTGGGGTCTGCTGCCCTGGATGCTGTGACGGCATCAGAGCA3′ (sense), 5′TGCTCTGATGCCGTCACAGCATCCAGGGCAGCAGACCCCACGCAGGAG3′(antisense);

### EL4-CCPGCC (1156–1205)

5′ CGGGGACGTGGCCAAGTGCTGCCCAGGGTGCTGCTTCATCATCTACCCGG 3′ (sense), 5′CCGGGTAGATGATGAAGCAGCACCCTGGGCAGCACTTGGCCACGTCCCCG3′(antisense).

Mutations were confirmed by sequencing (UMASS Medical School Nucleic Acid Facility) and mutagenic regions were subcloned back into the parental hDAT cDNA using the following restriction enzymes: PflMI/BstEII (EL2-CCPGCC), PflMI/ClaI (EL3-CCPGCC), BstEII/ClaI (EL4-CCPGCC).

### Cell Culture and Transfections

PC12 cells were from ATCC as previously described [22] and were cultured at 10% CO_2_ in high glucose DMEM supplemented with 5% horse serum, 5% bovine calf serum, 2 mM glutamine and 10^2^ U/ml penicillin-streptomycin. Cells were removed from the cultureware by spraying media directly onto the growth surface and triturating. Cells were transiently transfected either with Lipofectamine 2000 (Invitrogen), according to the manufacture's instructions, or by electroporation as previously described [23]. Briefly, 1.2×10^7^ cells were collected by centrifugation and resuspended in 0.75 ml electroporation buffer (137 mM NaCl, 5.0 mM KCl, 0.7 mM Na_2_HPO_4_, 6.0 mM glucose, 20 mM HEPES, pH 7.05) and were mixed with 18.2 µg of the indicated plasmids. Cells were electroporated at 300 mV, 500 µF, with an exponential decay protocol, using a GenePulser Xcell unit (Biorad, Hercules, CA) with a CE module and 4.0 mm cuvettes. Following 30 min recovery in PC12 media containing 3 mM EGTA, cells were plated onto poly-D-lysine coated plates as indicated. All transfected cells were assayed 48 hrs post-transfection.

### Surface Biotinylation and Immunoblotting

Cells were seeded in 6 well plates coated with 0.5 mg/ml poly-D-lysine 48 hours prior to performing assays. For surface biotinylation, cells were washed with phosphate buffered saline supplemented with 1.5 mM MgCl_2_, 0.2 mM CaCl_2_ (PBS^2+^) and surface proteins were covalently labeled biotin by incubating 2×15 min, 4°C with freshly prepared 1.0 mg/ml sulfo-NHS-SS-biotin (Pierce) prepared in PBS^2+^. Residual biotinylation reagent was quenched by washing cells with and incubating 2×15′, 4°C in PBS^2+^, 100 mM glycine. Cells were lysed in RIPA buffer (10 mM Tris, pH 7.4, 150 mM NaCl, 1.0 mM EDTA, 0.1% SDS, 1.0% Triton X 100, 1.0% sodium deoxycholate) containing protease inhibitors (1.0 mM PMSF, 1.0 µg/ml each leupeptin, aprotinin and pepstatin), 20 min, 4°C, cellular debris was cleared by centrifugation and protein concentrations were determined with the BCA protein assay (Pierce). Surface (biotinylated) proteins were separated from intracellular (non-biotinylated) proteins by streptavidin batch affinity chromatography using streptavidin-coupled agarose beads (Pierce), incubating overnight, 4°C with rotation. Non-biotinylated proteins in the supernatants were removed and concentrated by spin filtration using columns with 30 kDa molecular weight cutoff (Millipore). Beads bound to biotinylated proteins were washed three times with RIPA buffer and bound proteins were eluted in 2x Laemmli sample buffer by rotating 30 min, 25°C. Samples were separated on 10% SDS-PAGE gels and were transferred onto nitrocellulose for 1 hour, 100 V, 4°C. Blots were blocked with 5% nonfat dry milk in PBS/0.1% Tween-20 (PBS-T), 45 min, 25°C, and incubated with rat anti-DAT antibody (MAB369, Chemicon, 1∶1000) overnight, 4°C. Blots were washed with PBS-T and incubated with HRP-conjugated goat anti-rat antibody (Santa Cruz, 1∶5000), 45′, 25°C, following by washing in PBS-T. Blots were developed with Supersignal Dura (Pierce) and immunoreactive bands were detected with a VersaDoc imaging station (Bio-Rad). Non-saturating bands were quantified using Quantity One software and results were analyzed using GraphPad Prism software. Mature:immature protein ratios were calculated by summing mature band (90 kDa) densities in surface and intracellular fractions, and dividing by the immature band (56 kDa) density in the intracellular fraction.

### Immunocytochemistry and Microscopy

Transfected cells were seeded on glass coverslips coated with 1.0 mg/ml poly-D-lysine 48 hours prior to fixation and labeling. Cells were treated as indicated, rinsed in PBS and fixed in 4% paraformaldehyde prepared in PBS, 10 min, 25°C. Cells were blocked and permeabilized by incubating in blocking solution (PBS, 1% IgG/Protease-free BSA, 5% goat serum, 0.2% Triton-X-100), 30′, 25°C, followed by incubation with rat anti-DAT antibody (1∶2000 in blocking solution), 45 min, 25°C. Cells were washed with PBS and incubated with Alexa594-conjugated goat anti-rat antibody (1∶5000, Molecular Probes), 45 min, 25°C. Cells were washed with PBS, dried and mounted on glass slides with ProLong Gold (Molecular Probes). Immunoreactive cells were visualized as previously described [24] with a Zeiss Axiovert 200 M microscope using a 63X, 1.4 N.A. oil immersion objective and 0.4 µm optical sections were captured through the z-axis with a Retiga-1300R cooled CCD camera (Qimaging) using Slidebook 4.0 software (Intelligent Imaging Innovations). Z- stacks were deconvolved with a constrained iterative algorithm using measured point spread functions for each fluorescent channel using Slidebook 4.0 software. All images shown are single 0.4 µm planes through the center of each cell.

### [^3^H]DA Uptake Assays

Transfected cells were seeded onto 24 well plates coated with 0.5 mg/ml poly-D-lysine and [^3^H]DA uptake was measured 48 hours post transfection as previously reported [23], [25]. Briefly, cells were rinsed and pre-incubated in KRH buffer (120 mM NaCl, 4.7 mM KCl, 2.2 mM CaCl_2_, 1.2 mM MgSO_4_, 1.2 mM KH_2_PO_4_, 0.18% glucose, 10 mM HEPES, pH 7.4) at either for 37°C for 30 min with the indicated drugs. 100 nM desipramine was included in all wells to block endogenous NET activity. Uptake was initiated by adding 1.0 µM [^3^H]DA (dihydroxyphenylethylamine 3,4-[ring-2,5,6,-^3^H], Perkin Elmer) containing 10^−5^ M pargyline and 10^−5^ M ascorbic acid. Assays proceeded for 10 min (37°C) and were terminated by rapidly washing cells with ice-cold KRH buffer. Cells were solubilized in scintillation fluid and accumulated radioactivity was determined by liquid scintillation counting in a Wallac Microbeta scintillation plate counter. Non-specific uptake was defined in the presence of 10 µM GBR12909 and averaged <5% of total counts measured. Data analysis was performed using Microsoft Excel and GraphPad Prism Software.

## Results

**Figure 1:**
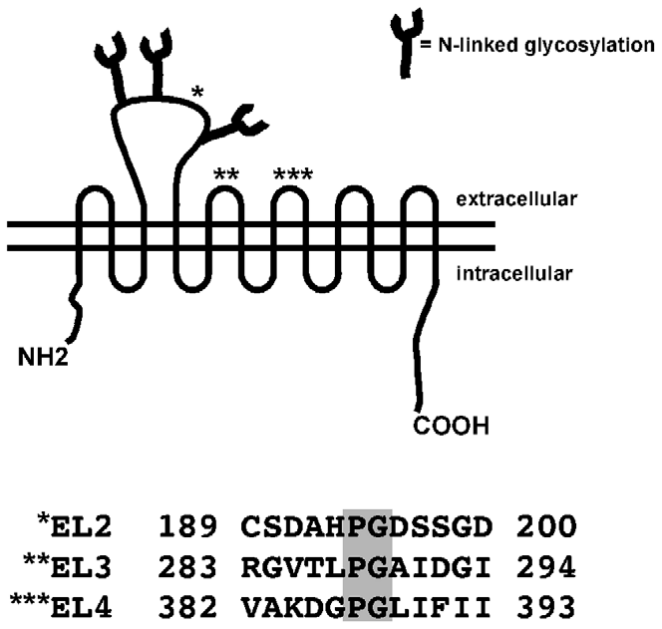
Schematic of DAT with target sites for tetracysteine mutagenesis. *Top:* DAT model. Asteriks indicate extracellular Pro-Gly residues targeted for tetracysteine mutagenesis. *Bottom:* DAT sequences spanning across the Pro-Gly tetracysteine target sites. Pro-Gly dipeptides are highlighted in the shaded boxes.

**Figure 2:**
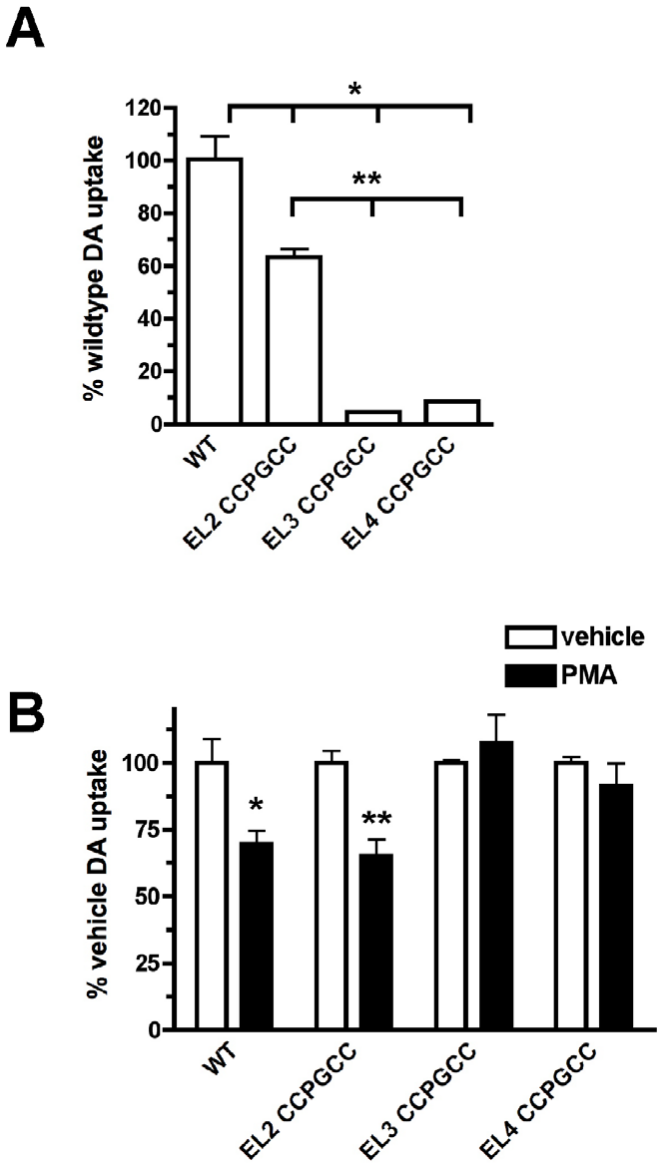
DA uptake and PKC-mediated downregulation are preserved when a tetracysteine motif is introduced into EL2, but not EL3 or EL4. *DA uptake assay*. Cells were transfected as described in *Methods* and [^3^H]DA uptake was assessed 48 hours post transfection. **A.** Introduction of tetracysteine motifs decreases DA uptake. Data are expressed as % wildtype activity ±S.E.M. *Significantly different from wildtype (p<.01, One-way ANOVA with Tukey's multiple comparison test, n = 3), **Significantly different from EL2-CCPGCC DAT (p<.001, One-way ANOVA with Tukey's multiple comparison test, n = 3). **B.** EL2-CCPGCC DAT is acutely downregulated by PKC activation, whereas EL3- and EL4-CCPGCC are not. Cells were treated with either vehicle or 100 nM PMA, 30 min, 37°C and [^3^H]DA uptake was measured. Data are expressed as % vehicle-treated DA uptake ±S.E.M. for each construct. *Significantly different from vehicle treated (p<.05, Student's t test, n = 3), **significantly different from vehicle treated (p<.02, Student's t test, n = 3).

Protein labeling with biarsenic dyes requires the presence of a tetraCys motif, optimally flanking a Pro-Gly dipeptide. As depicted in Figure 1, DAT encodes three predicted extracellular Pro-Gly sequences at residues 194, 288 and 387. We took advantage of these intrinsic Pro-Gly residues and mutated the 2 upstream and downstream residues to cysteines to generate three DAT constructs with CCPGCC sequences in either extracellular loops 2, 3 or 4 (termed DAT EL2-CCPGCC, EL3-CCPGCC and EL4-CCPGCC, respectively). We first tested whether the mutant DATs retained DA transport function as compared to wildtype DAT. As seen in Figure 2A, all three tetraCys mutants exhibited significantly reduced DA uptake as compared to wildtype DAT (p<.01). However, DAT-EL2-CCPGCC retained 63.5% of wildtype activity, which was significantly greater than both DAT-EL3-CCPGCC and DAT-EL4-CCPGCC (p<.001). We next tested whether any of the DAT tetraCys mutants were capable of undergoing PKC-mediated downregulation. Following treatment with 100 nM PMA, 30′, 37°C, wildtype DAT function decreased by 30.7±2.3% (Fig. 2B). Similarly, PKC activation decreased DAT-EL2-CCPGCC activity by 29% (Not significantly different from wild type, p = 0.77, Student's t test, n = 3). In contrast, PKC activation had no effect on either DAT-EL3-CCPGCC or DAT-EL4-CCPGCC (Fig. 2B). These data suggest that introduction of a tetraCys motif into the DAT EL2 results in a functional DAT that undergoes PKC-mediated downregulation, whereas conversion of residues in EL3 and EL4 is not well tolerated by DAT.

**Figure 3:**
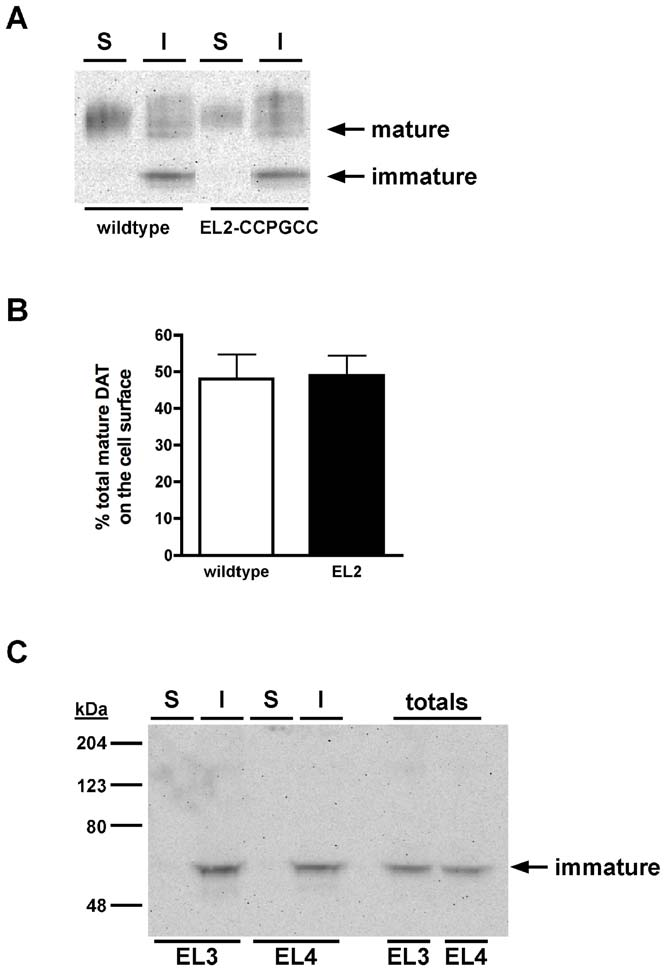
The presence of a tetracysteine in DAT EL2 does not alter DAT maturation or surface expression. *Surface biotinylation assay*. Cells were transfected with the indicated DAT constructs and cell surface proteins were biotinylated and isolated from intracellular proteins as described in *Methods*. **A.** Representative immunoblot displaying surface (S) and intracellular (I) wildtype and EL2-CCPGCC DAT. Mature (90 kDa) and immature (56 kDa) species are indicated. **B.** Averaged data. Data are expressed as %total mature DAT on cell surface ±S.E.M. (n = 4). **C.** Representative immunoblot displaying surface (S) and intracellular (I) EL3-CCPGCC and EL4-CCPGCC DAT. Total lysates are displayed in the far right hand lanes. Note that no mature protein is detected. Immature species (56 kDa) are indicated.

We next used cell surface biotinylation to test whether the tetracysteine DATs exhibited maturation and cell surface expression comparable to wildtype DAT. As seen in Figure 3A, surface and intracellular proteins are readily detectable for both wildtype and EL2-CCPGCC DATs, and no significant difference between the %total mature DAT on the cell surface was detected (Fig. 3B, 48.1±6.7% (wildtype) vs. 49.0±5.4% (EL2-CCPGCC), p = .92, Student's *t* test, n = 4). In contrast, neither EL3 nor EL4 were detected at the cell surface, nor was any mature protein detected, suggesting that these mutants fail to progress through the biosynthetic pathway (Fig. 3C). We also measured the ratios of mature (90 kDa) to immature (56 kDa) DAT as an index of how readily EL2-CCPGCC DAT progresses through the biosynthetic pathway. Wildtype DAT exhibited mature:immature protein ratio of 1.66±0.22, whereas EL2-CCPGCC DAT had a trend for less mature:immature protein (0.99±.17), but which was not significantly different from wildtype (p = .07, Student's t test, n = 4), suggesting that EL2-CCPGCC DAT is processed through the biosynthetic pathway comparably to wildtype DAT.

**Figure 4:**
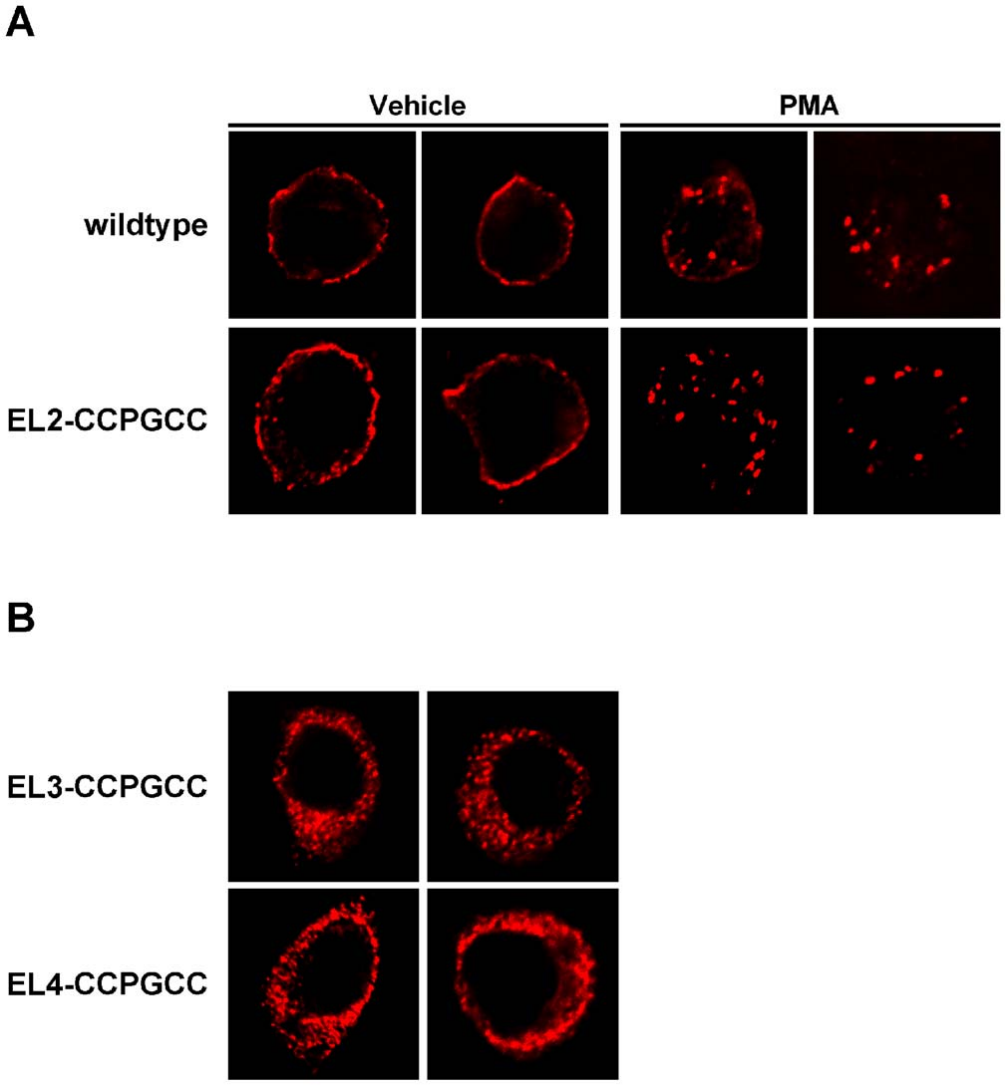
Cellular distribution of tetracysteine mutant DATs. EL2-CCPGCC DAT is robustly expressed at the cell surface and internalizes in response to PKC activation. *Immunofluorescence microscopy*. Cells were transfected with the indicated constructs and were fixed and stained with rat anti-DAT antibodies as described in *Methods*. A representative panel of deconvolved images is presented. A single plane through each cell center is shown. **A**. Effect of PKC activation on EL2-CCPGCC DAT. Prior to fixation, cells were treated with either vehicle or 1 µM PMA (37°C, 30 min). **B**. Cellular distribution of EL3-CCPGCC and EL4-CCPGCC DAT. Note the lack of either protein at the cell surface.

We next used fluorescence microscopy to examine the cellular distribution of the tetracysteine-tagged DATs as compared to wildtype DAT in transiently transfected PC12 cells. Wildtype DAT staining presented as an intense ring at the cell perimeter, and PKC activation resulted in robust DAT redistribution to intracellular puncta (Fig. 4A). Similarly, EL2-CCPGCC DAT staining was primarily at the cell perimeter (Fig. 4A), although some intracellular staining was apparent. Moreover, PKC activation resulted in pronounced surface losses of EL2-CCPGCC DAT, similar to that observed for wildtype and consistent with our uptake results demonstrating functional downregulation of EL2-CCPGCC DAT. We also expressed EL3, EL4 mutants and visualized their cellular distribution. Neither EL3 nor EL4 were present at the cell perimeter, and a diffuse perinuclear signal was detected (Fig. 4B). These results are consistent with our uptake and surface biotinylation results suggesting that EL3 and EL4 fail to mature and traffic to the cell surface and that these mutations are not well tolerated by DAT.

## Discussion

In the current study, we aimed to introduce tetraCys residues in DAT extracellular domains that intrinsically encode Pro-Gly dipeptide sequences at DAT amino acid positions 194, 288 and 387. The CCPGCC sequence is the optimal sequence to facilitate biarsenic dye binding, and once bound confers the proper dye conformation for fluorescence emission [19], [21]. Converting the four residues flanking the Pro-Gly at amino acid positions 194–195 yielded a DAT that retained DAT surface expression comparable to wildtype. Moreover, EL2-CCPGCC DAT exhibit robust DA uptake, albeit somewhat reduced as compared to wildtype, and retained sensitivity to PKC-mediated downregulation, suggesting that this mutant may be amenable to labeling with biarsenic dyes for live imaging studies of DAT trafficking. A recent study indicated that inserting a tetraCys motif at the DAT amino terminus and at position 511 were also well tolerated [26]. However, these are both located on intracellular domains that would require use of a membrane permeant biarsenic dye that would have access to the entire cellular DAT population and would not exclusively label the DAT surface residents. It is also interesting to note that although we reproducibly observe PKC-mediated DAT downregulation in PC12 cells, a recent study by Ericksen et al reports lack of PKC-mediated downregulation in primary ventral midbrain neurons prepared from P2 rat pups [16]. This conflicts with previous studies that report PKC-mediated DAT downregulation in adult rat striatal synaptosomes [27], and suggests that either the developmental stage of the dopaminergic neuron or the primary culture environment may influence the ability of DAT to undergo PKC-mediated downregulation.

While our data demonstrate that an extracellular tetracysteine-tag is well tolerated by DAT, they do not demonstrate whether this DAT construct can be labeled with biarsenic dyes. We made several attempts to label EL2-CCPGCC DAT using Lumio brand (Invitrogen) FlAsH reagent, using both standard approaches as well as modified protocols that use extended washes with BAL reagent to reduce non-specific FlAsH binding. All of our labeling attempts resulted in a high degree of non-specific labeling of non-transfected cells, as well as cells expressing either wildtype or EL2-CCPGCC DAT (not shown). Problems with non-specific FlAsH binding have been described [28], [29] and particular issues have been raised regarding the utility and specificity of the commercially available Lumio form of FlAsH [30]. Thus, while our EL2-CCPGCC DAT is a viable DAT form, advances in FlAsH labeling may be required in order to determine the utility of this mutant in future trafficking studies.

DAT residues 192–197 are located between the second and third glycosylation sites of the large second extracellular loop of the protein. This region appears to tolerate amino acid substitution well, as previous studies reported that an extracellular HA epitope was successfully engineered across residues 193–203 [31]. The HA epitope at this locus is accessible for extracellular antibody labeling, suggesting that the much smaller biarsenic dye would also have access to this DAT region. Cysteine conversion of EL3 and EL4 residues flanking the Pro-Gly at positions 288–289 and 387–388, respectively, yielded CCPGCC DAT mutants that were not highly functional. Interestingly, a previous mutagenesis study in which a cysteine-depleted DAT was generated indicated that mutating an existing cysteine in EL3 at position 306, just downstream of the EL3-CCPGCC DAT, resulted in the expression of mature DAT protein [32] that is fully functional [33]. Cys306 is positioned close to the sixth transmembrane segment, suggesting that this region may be more tolerable to structural perturbation.

In summary, we have characterized a functional DAT mutant that encodes a tetraCys motif on the extracellular DAT domain EL2. The ability to introduce cysteines at this locus may have future utility both for NTSEA reagent labeling, as well as for biarsenic dye labeling for live imaging studies. Future studies will directly examine the capacity of this mutant to bind to biarsenic dyes.
